# Association between Proinflammatory Markers, Leukocyte–Endothelium Interactions, and Carotid Intima–Media Thickness in Type 2 Diabetes: Role of Glycemic Control

**DOI:** 10.3390/jcm9082522

**Published:** 2020-08-05

**Authors:** Aranzazu Martinez de Marañón, Francesca Iannantuoni, Zaida Abad-Jiménez, Francisco Canet, Pedro Díaz-Pozo, Sandra López-Domènech, Ildefonso Roldán-Torres, Carlos Morillas, Milagros Rocha, Víctor M. Víctor

**Affiliations:** 1 Service of Endocrinology and Nutrition, University Hospital Doctor Peset, Foundation for the Promotion of Health and Biomedical Research in the Valencian Region (FISABIO), 46017 Valencia, Spain; amardema@alumni.uv.es (A.M.d.M.); franian@alumni.uv.es (F.I.); zaiaji@alumni.uv.es (Z.A.-J.); francisco.canet.1994@gmail.com (F.C.); pedrodpas2@gmail.com (P.D.-P.); Sandra.Lopez@uv.es (S.L.-D.); carlos.morillas@uv.es (C.M.); 2 Service of Cardiology, University Hospital Doctor Peset, Foundation for the Promotion of Health and Biomedical Research in the Valencian Region (FISABIO), 46017 Valencia, Spain; il.roldant@comv.es; 3 Centro de Investigación Biomédica en Red (CIBERehd)—Department of Pharmacology, University of Valencia, 46010 Valencia, Spain; 4 Department of Physiology, University of Valencia, 46010 Valencia, Spain

**Keywords:** type 2 diabetes, glycated hemoglobin, carotid intima–media thickness, inflammation, endothelial function

## Abstract

Glycated hemoglobin monitorization could be a tool for maintaining type 2 diabetes (T2D) under control and delaying the appearance of cardiovascular events. This cross-sectional study was designed to assess the role of glycemic control in modulating early-stage markers of cardiovascular complications. One hundred and eight healthy controls and 161 type 2 diabetic patients were recruited and distributed according to their glycemic control, setting the threshold at 6.5% (good control). Biochemical and anthropometrical parameters were registered during the initial visit, and peripheral blood was extracted to obtain polymorphonuclear cells and analyze inflammatory markers, adhesion molecules, leukocyte–endothelium interactions, and carotid intima–media thickness. Correlations between these parameters were explored. We found that inflammatory markers and adhesion molecules were augmented in type 2 diabetic subjects with poor glycemic control. Polymorphonuclear leukocytes interacted more with the endothelium in the diabetic population, and even more significantly in the poorly controlled subjects. In parallel, carotid intima–media thickness was also increased in the diabetic population, and the difference was greater among poorly controlled subjects. Finally, correlation measurement revealed that carotid intima–media thickness was related to glycemic control and lipid metabolism in diabetic patients. Our results suggest that glycemic control delays the onset of cardiovascular comorbidities in diabetic subjects.

## 1. Introduction

Type 2 diabetes (T2D) is currently one of the most prevalent metabolic diseases, affecting around 500 million people. Its incidence has doubled since 1980 [1,2], increasing health expenditure because of T2D itself and due to its derived complications [1,2,3,4]. In fact, cardiovascular diseases (CVD) are the leading cause of death among type 2 diabetic subjects [2,5], being caused mainly by advanced atherosclerosis, which can be delayed or prevented by early and maintained glycemic control. One of the key markers of glycemic control is glycated hemoglobin (HbA_1c_), and reducing its levels is a primary goal of diabetic treatment [6,7,8]. In fact, several studies have demonstrated that sustaining HbA_1c_ below 6.5% reduces the incidence of macro- and microvascular comorbidities [7,9,10,11,12,13].

T2D is associated with a proinflammatory background, caused by high circulating glucose, accumulation of advanced glycation end products (AGEs), glycation of hemoglobin, alteration of lipid metabolism in adipose tissue, and other metabolic alterations that favor a proinflammatory state in peripheral blood [14,15,16]. If sustained for long periods, all of these modifications promote the production by tissue of a wide array of proinflammatory cytokines such as Interleukin-6 (IL6) and Tumor Necrosis Factor alpha (TNFα), as well as reactive oxygen species (ROS) [15,16,17,18,19,20], specially by mitochondria. In short, hyperglycemia and hyperlipidemia trigger Nod-Like Receptor Protein NLRP inflammasome activation, TNFα synthesis, and the production of mitochondrial and nonmitochondrial ROS [21,22,23]. This induces Nuclear Factor Kappa B (NFκB) activation and inflammatory cytokine expression, mostly through thioredoxin-related protein action [24,25,26,27]. Moreover, lipids can react with ROS and amplify the proinflammatory cascade [28]. This results in a vicious cycle of cell death and greater inflammation [29]. This ROS–inflammation axis has been studied in a wide array of inflammatory-based diseases, such as cardiac alterations [30,31,32], bone and joint diseases [33,34,35], neuronal and cerebral dysfunctions [28,29,36], bacterial infection [37], liver diseases [38], respiratory alterations [39,40] and cancer [41]. Furthermore, in T2D, the continuous presence of proinflammatory molecules causes diverse endocrine effects on the vasculature, and contributes to the development of micro- and macrovascular pathologies such as carotid atherosclerosis [21]. Together, the sustained increase of ROS production and the rise in inflammation have an important effect on the development of diabetic atherosclerosis [22].

Immune cells are also activated in T2D, producing more proinflammatory and adhesion molecules [15,16,42,43]. As explained previously, circulating proinflammatory molecules produced by chronic hyperglycemia and hyperlipidemia can activate leukocytes and the endothelium [44,45]. In this state, immune cells interact with the endothelium, infiltrating the inner layers of tissues and intensifying the inflammation [15,44,45]. There are different epidemiologic studies describing how an increased leukocyte count is a risk factor for the progression of carotid atherosclerosis and cardiovascular events [46,47,48]. Proinflammatory factors also favor the development of the atherosclerotic plaque, as demonstrated by several studies [49,50,51,52]. In fact, atherosclerosis represents the culmination of continued subclinical inflammation, and is one of the main causes of cardiovascular comorbidities [6,23,53,54,55]. Worryingly, atherosclerosis is often asymptomatic for decades before clinical manifestations appear, and is termed subclinical atherosclerosis during this period [6,56,57].

Carotid intima–media thickness (CIMT) is a biomarker of subclinical atherosclerosis [58,59]. Measurement of the CIMT by B-mode ultrasound has been shown to be suitable for evaluating the early stages of atherosclerosis [57,60,61] and to be an indicator of CVD [62,63,64]. Different studies have described a rise of CIMT in T2D [63,65,66] and metabolic syndrome [67,68].

The aim of this study was to explore the potential involvement of glycemic control in inflammation, adhesion molecules, leukocyte–endothelium interactions, and the CIMT in T2D patients compared with a healthy control population.

## 2. Experimental Section

### 2.1. Human Subjects

This study was carried out in 269 subjects, specifically, 161 T2D patients and 108 healthy controls recruited from the Service of Endocrinology and Nutrition of University Hospital Doctor Peset (Valencia, Spain) until June 2019 and adjusted for age and sex. The time that these patients had suffered from T2D, the presence of comorbidities, and their drug prescriptions are specified in Supplementary data (Tables S1–S3). The subjects signed a written informed consent form and protocols were approved by our hospital’s Ethics Committee for Clinical Investigation (ID: 98/19), in line with the ethical principles of the Helsinki declaration. T2D patients were diagnosed following the American Diabetes Association (ADA) indications, and presence of morbid obesity, insulin treatment, or any autoimmune, hematological, malignant, infectious, organic, or inflammatory disease represented the exclusion criteria.

### 2.2. Sample Collection

Venous blood samples were obtained from the antecubital vein in fasting conditions. Weight (kg), height (m), body mass index (BMI; kg/m2), systolic and diastolic blood pressure (SBP/DBP; mmHg), and waist circumference (cm) were assessed previous to the blood extraction.

### 2.3. Laboratory Tests

Serum was isolated from the blood by centrifugation for 10 min at 1500g and 4ºC. Fasting glucose, cholesterol, and triglycerides were determined by an enzymatic method. High-density lipoprotein cholesterol (HDL-c) levels were measured with a Beckman LX-20 autoanalyzer (Beckman Coulter, La Brea, CA, USA) using a direct method. Low-density lipoprotein cholesterol (LDL-c) was determined with Friedewald’s formula. An immunochemiluminescence assay was employed to determine insulin levels. Homeostatic Model Assessment of insulin resistance HOMA-IR index [fasting insulin (μU/mL) × fasting glucose (mg/dl)/405] was calculated to estimate insulin resistance (IR). Percentage of HbA_1c_ was determined with an automatic glycohemoglobin analyzer (Arkray, Inc., Kyoto, Japan). Apolipoproteins were measured with an electroimmunoassay. High-sensitivity C-reactive protein (hsCRP) was analyzed employing an immunonephelometer (Behring Nephelometer II, Dade Behring, Inc., Newark, DE, USA).

### 2.4. Leukocyte Isolation

In this assay, polymorphonuclear leukocytes (PMNs) were isolated from heparinized whole blood by the following protocol: the blood was mixed with 1:2 volumes of dextran solution (3% in NaCl 0.9%; Sigma Aldrich, MO, USA) and incubated for 45 min. The supernatant was then poured over Ficoll-Hypaque (GE Healthcare, Uppsala, Sweden) and centrifuged at 650 g for 25 min. The resulting pellet was lysed to remove the remaining erythrocytes with lysis buffer (5 min at room temperature) and centrifuged at 240 g. Pellets containing leukocytes were then washed twice and resuspended in Hank’s balanced salt solution (HBSS; Sigma Aldrich, MO, USA). This cellular suspension was employed to perform the leukocyte–endothelium interaction assay.

### 2.5. Soluble Cytokines and Adhesion Molecule Assay

Intercellular adhesion molecule 1 (ICAM-1), vascular cell adhesion molecule-1 (VCAM-1), P-selectin, IL-6, and TNFα were analyzed in serum samples with a Luminex 200 flow analyzer system (Millipore, Austin, TX, USA). In brief, specific antibodies covered the color-coded microbead, and detection was performed with biotinylated secondary antibody and streptavidin-PE conjugate. The fluorescence of each individual microbead was analyzed with the Luminex XMap instrument. This method allows multiple cytokines in the same sample to be analyzed with a high specificity ad sensitivity. The TNFα detection range was between 1750 and 0.43 pg/mL; that of IL-6 is 750 to 0.18 pg/mL; that of VCAM-1 is 500 to 0.122 ng/mL; that of ICAM-1 is 350 to 0.085 ng/mL; and that of P-selectin is 1000 to 0.122 ng/mL. The intra-assay %CV is <5% for TNFα and IL-6 and <15% for ICAM-1, VCAM-1, and P-selectin. The interassay %CV is <20% for IL-6, ICAM-1, VCAM-1, and P-selectin and <15% for TNFα.

### 2.6. Static Cytometry Measurements

Mitochondrial ROS production was evaluated employing a MitoSOX (Thermo Fisher Scientific, Waltham, MA, USA) fluorescent probe. A fluorescence microscope (IX81; Olympus Corporation, Shinjuku-ku, Tokyo, Japan) with automated static cytometry software (ScanR, Olympus, Munich, Germany), which measures the fluorescence emission per individual cell, was also used. In brief, the protocol consisted of seeding PMN, extracted as previously specified, in 48-well plates and allowing them to adhere to the well surface. MitoSOX and DAPI (Sigma Aldrich, MO, USA) were then added to the well at a final concentration of 0.1µM, for 20 min. After washing the cells twice with HBSS, fluorescence was measured and MitoSOX emission data relativized to DAPI emission data for each cell. PMN data were relativized with an internal control for all the experiments. All experiments were performed in duplicate, and 16 images per well were measured.

### 2.7. PMN–Endothelium Interaction Assay

An aliquot of 1.2 mL PMNs, isolated as previously described [69], with a density of 10^6^ cells/mL in complete Roswell Park Memorial Institute medium RPMI, was employed for this assay. Primary cultures of human umbilical cord endothelial cells (HUVEC) were prepared as reported in [69]. In this assay, the PMN aliquot was perfused across the endothelial monolayer at a speed of 0.3mL/min over a 5 min period, and the process was recorded. Rolling PMNs were considered to be those rolling for at least 1 min. Velocity was assessed by determining the time in which 15 rolling PMNs covered a distance of 100 μm. Adhesion was analyzed by counting the number of PMNs adhering to the endothelium for at least 30 s in 5 different fields.

### 2.8. Assessment of Carotid Intima–Media Thickness (CIMT)

Carotid thickness was evaluated following the American Echocardiography Association’s guidelines. Healthy subjects and T2D patients were told to attend the Cardiology Service of the Dr. Peset Hospital 7-10 days after the blood extraction in order to evaluate carotid intima–media thickness. This measure has a diagnostic value because of its positive correlation with risk factors and with the prevalence of cardiovascular and cerebrovascular disease. The evaluation was performed by placing the head of the patients at 45º with respect to the body longitudinal axis. Some subjects were dropped from the study due to clinical or schedule reasons.

Carotid sonography was performed with a single ultrasound machine Aloka 5500 (Hitachi Aloka, Tokyo, Japan) equipped with a 7.5 MHz sector scanner probe. Baseline and follow-up studies were performed in a standard fashion by a single specialist physician who was specifically trained to perform the examination and was blinded to the treatment group. All images were electronically stored. Measurements corresponded with the 1 cm segment proximal to the dilation of the carotid bulb, and were always performed in plaque-free segments. For each patient, three measurements were performed for both sides of the anterior, lateral, and posterior projections of the far wall, and readings were then averaged. An independent observer, who was blinded to the treatment group and trained to interpret the CIMT images, performed an off-line analysis of B-mode ultrasound images. Paired CIMT measurements in the same arteries showed a high degree of reproducibility, with a mean difference in CIMT of 0.020 mm, and an intraclass correlation coefficient of 0.97 (*p* < 0.001). CIMT regression was defined as a decrease of >0.020 mm in mean CIMT at 12 months.

### 2.9. Statistical Analysis

All data were analyzed with SPSS 17.0 software (SPSS Statistics Inc., Chicago, IL, USA). Values are expressed as mean and standard deviation (SD) for parametric data, while the median (25th–75th percentiles) is presented for nonparametric data. Bar graphs show mean and standard error of the mean (SEM) in the figures. Multivariate lineal analysis was performed to check the influence of BMI and age on the other dependent variables.

Correlation analysis was performed with the Spearman formula, and the linear regression coefficient was also calculated. Graphs were plotted with GraphPad Prism 4.0 (GraphPad, La Jolla, CA, USA).

Multivariate linear analysis was performed in order to eliminate the influence of BMI and age on the variables of interest. Normality of the data sets was assessed by Kolmogorov–Smirnov test. In the case of the variables with normally distributed data, the groups were compared with a Student’s *t*-test, while a Mann–Whitney U test was employed for non-normally distributed ones, and the Chi-Square test for proportion of frequencies. Study groups were compared using one-way analysis of variance (ANOVA) followed by a Bonferroni post hoc test. Differences were considered to be significant when *p* < 0.05, applying a confidence interval of 95% in every comparison. Graphs were plotted with GraphPad Prism 4.0 (GraphPad, La Jolla, CA, USA).

## 3. Results

### 3.1. Anthropometric and Biochemical Parameters

The study population was initially divided into healthy controls (108) and T2D patients (161) following the diagnostic criteria of the ADA. Diabetic patients were divided into two populations depending on their glycemic control, which was represented by their levels of HbA_1c_. The set threshold was 6.5, in line with ADA criteria [7]. Table 1 confirms that both diabetic populations had typical hallmarks, with significant differences in glucose (*p* < 0.01), HbA1c (*p* < 0.01), and HOMA index (*p* < 0.01). Moreover, significant differences in glucose levels (*p* < 0.001) and HOMA index (*p* < 0.001) were found between the T2D HbA_1c_ > 6.5 group and the T2D HbA_1c_ ≤ 6.5 group. Our T2D populations also displayed features such as greater waist diameter (*p* < 0.01), increased waist-to-hip ratio (*p* < 0.01), higher HOMA index (*p* < 0.01), and altered lipid metabolism parameters, with increased VLDL and triglyceride levels (*p* < 0.01), Ct/HDL (*p* < 0.01), and AIP (*p* < 0.01) and lower levels of HDL-c (*p* < 0.01). Total cholesterol, LDL cholesterol, and non-HDL cholesterol levels remained unchanged in the T2D HbA_1c_ ≤ 6.5% group. However, total and LDL cholesterol were significantly reduced (*p* < 0.01) in the T2D HbA_1c_ > 6.5 group, possibly due to the hypolipemiant treatment. Regarding apolipoproteins, ApoA1 was significantly lower in T2D patients with respect to healthy controls (*p* < 0.01) and the difference was even more significant in the HbA_1c_ > 6.5% group (*p* < 0.001). ApoB levels did not change. Interestingly, the ApoB/ApoA ratio significantly increased in the T2D HbA_1c_ > 6.5 population (*p* < 0.01).

### 3.2. Inflammation Markers

**Figure 1:**
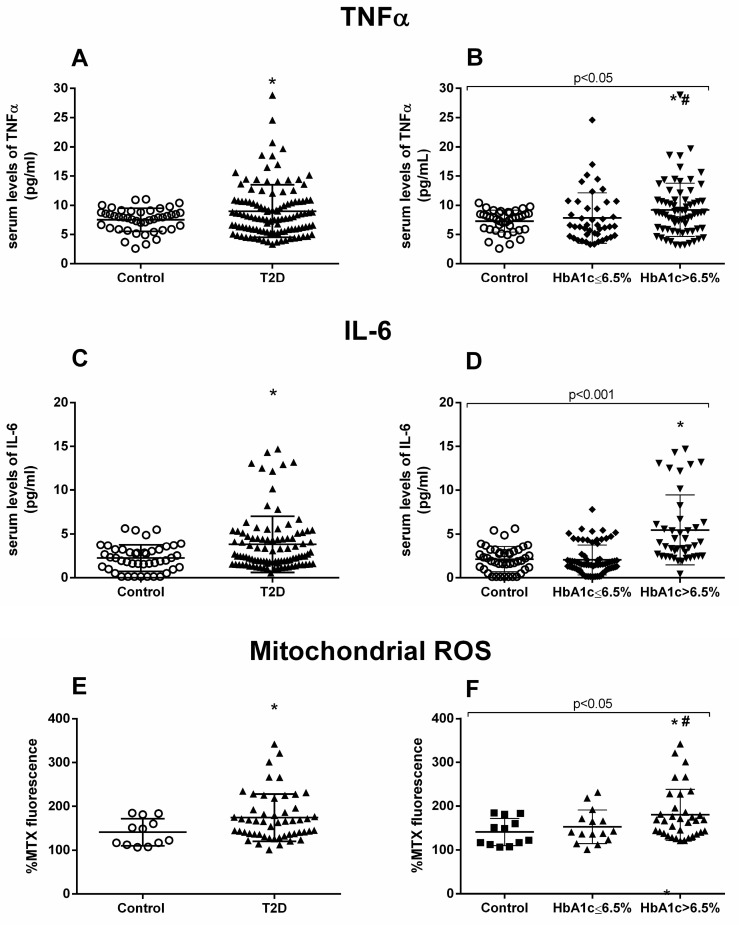
**TNFα, IL-6, and mtROS measurements.** Serum levels of proinflammatory cytokines TNFα (**A**,**B**) and IL-6 (**C**,**D**), and fluorescence levels of mtROS (**E**,**F**). Differences between control and T2D groups (**A**,**C**,**E**) or between control, well-controlled (HbA_1c_ ≤ 6.5%) and poorly controlled diabetic groups (HbA_1c_ > 6.5%) (**B**,**D**,**F**) are shown. Statistical analysis was performed using a t-test to compare two groups, and using ANOVA with Bonferroni post-test for three groups. *p < 0.05 vs. control; # *p* < 0.05 vs. T2D HbA1c < 6.5%.

A hyperglycemic scenario is usually accompanied by an increase in subclinical inflammation levels. We analyzed some relevant proinflammatory markers in our cohort of patients and their respective controls. The T2D group showed a significant increase in TNFα levels compared to the control group (*p* = 0.047) (Figure 1A). When we distributed the T2D population based on HbA_1c_, an increase was preserved in the T2D HbA_1c_ > 6.5 group (*p* = 0.014) (Figure 1B). Another relevant cytokine, IL-6, was doubled in T2D subjects (*p* = 0.019) (Figure 1C) and, as occurred with TNFα, the increase was associated with T2D HbA_1c_ >6.5% (*p* = 0.015) (Figure 1D).

Moreover, we evaluated mtROS production, and the results showed a significant rise in mtROS in the T2D population (*p* = 0.045) (Figure 1E), which was more pronounced among the poorly controlled population (*p* = 0.038) (Figure 1F). Poorly controlled T2D patients also had significantly higher levels of mtROS than their well-controlled counterparts (*p* = 0.041) (Figure 1F).

### 3.3. PMN–Endothelium Interactions

**Figure 2:**
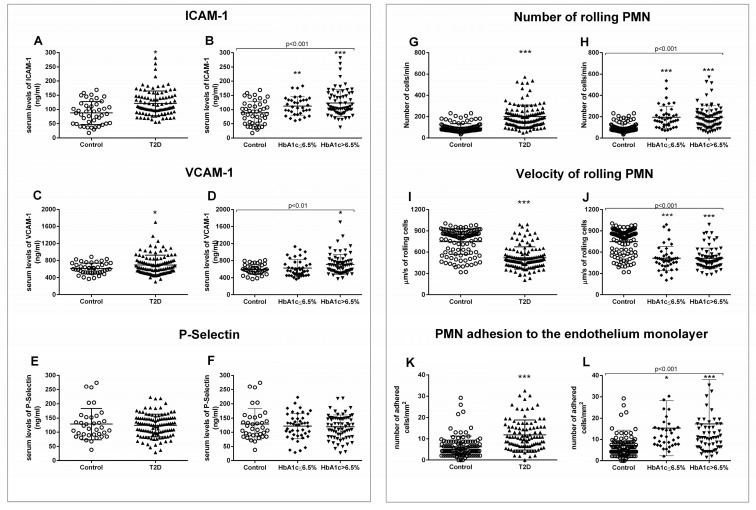
**Serum levels of soluble adhesion molecules and measurement of PMN–endothelium interactions**. Differences in adhesion molecules between control and T2D groups (**A**,**C**,**E**) or between control, well-controlled diabetics (HbA_1c_ ≤ 6.5%), and poorly controlled diabetics (HbA_1c_ > 6.5%) (**B**,**D**,**F**) are shown. The number of rolling cells (**G**,**H**), their velocity (**I**,**J**), and the adhesion of these cells to the endothelial monolayer (**K**,**L**) were analyzed. Differences between control and T2D groups (**G**,**I**,**K**) or between control, well-controlled diabetics (HbA_1c_ ≤ 6.5%), and poorly controlled diabetic groups (HbA_1c_ > 6.5%) (**H**,**J**,**L**) are shown. Statistical analysis was performed by means of a t-test to compare two groups, and using ANOVA with Bonferroni post-test for three groups. * *p* < 0.05; ** *p* < 0.01; *** *p* < 0.001 vs. control.

The generalized state of inflammation during T2D entails the activation of immune cells, which, in an active state, are more prone to attach to the endothelium and infiltrate through to the inner layers of the organs. Thus, we analyzed serum levels of adhesion molecules such as ICAM-1, VCAM-1, and P-selectin, some of the main players of leukocyte–endothelium interactions. As can be seen in Figure 2, T2D patients displayed higher levels of ICAM-1 (*p* = 0.016) (Figure 2A) and VCAM-1 (*p* = 0.027) (Figure 2C), but not of P-selectin. The increase in ICAM-1 was already significant in the well-controlled diabetic population (*p* = 0.006) (Figure 2B), and was enhanced in the poorly controlled diabetic population (*p* < 0.001). In addition, VCAM-1 was significantly higher in T2D subjects with HbA_1c_ > 6.5% (*p* = 0.005) (Figure 2D).

For assessing PMN–endothelium cell interactions directly, we performed an in vitro adhesion assay with leukocytes from T2D patients and their respective controls. Leukocyte count was slightly higher but within the normal range in T2D patients. This could reflect the subclinical inflammation level characteristic of T2D (Supplementary Table S4). Rolling number, rolling velocity, and adhesion to the endothelial monolayer were assessed. We obtained a higher number of rolling cells in T2D patients (*p* < 0.001) (Figure 2G), accompanied by a lower velocity of these cells (*p* < 0.001) (Figure 2I) and a higher level of adhesion to the endothelial monolayer (*p* < 0.001) (Figure 2K). These differences remained when we separated the T2D population depending on its glycemic control status (*p* < 0.001) (Figure 2H,J,L). PMN rolling (*p* < 0.001) and adhesion (*p* < 0.05) were increased, while rolling velocity was decreased (*p* < 0.001) in well-controlled diabetic subjects. These significant differences were sustained in poorly controlled diabetic subjects, and were more significant in the case of cell adhesion (*p* < 0.001). These differences can be assessed in the Supplementary Videos (Supplementary Videos S1–S3), in which representative videos of each experimental group have been attached (Supplementary data).

### 3.4. Carotid Intima–Media Thickness Measurements

**Figure 3:**
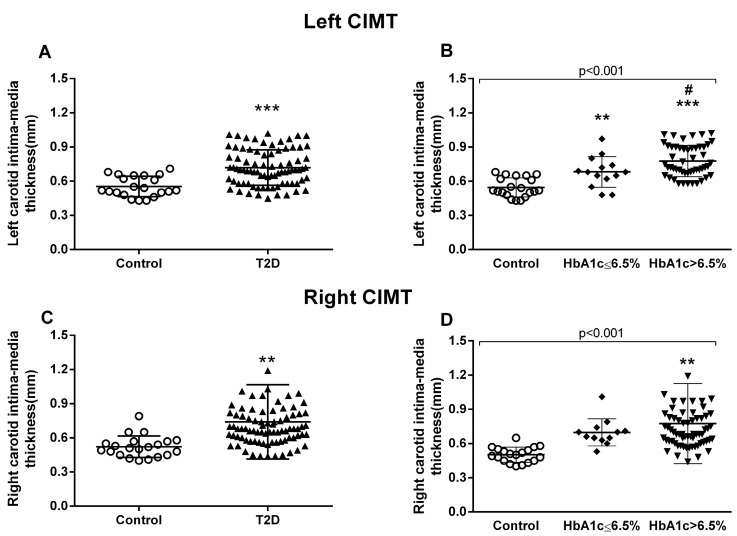
**Measurement of carotid intima–media thickness (CIMT).** Left carotid (**A**,**B**) and right carotid (**C**,**D**) were analyzed. Differences between control and T2D groups (**A**,**C**) or between control, well-controlled (HbA_1c_ ≤ 6.5%), and poorly controlled diabetic groups (HbA_1c_ > 6.5%) (**B**,**D**) are shown in the graphs. Statistical analysis was performed by means of a t-test to compare two groups, and using ANOVA with a Bonferroni post-test for three groups. ** *p* < 0.01; *** *p* < 0.001 vs. control group; # *p* < 0.05 vs HbA_1c_ ≤ 6.5% group.

The proinflammatory environment seen in our diabetic patients and the increase in leukocyte–endothelium interactions could represent a rise in the incidence of macro- and microvascular complications. Therefore, we next explored CIMT. All the patients underwent carotid echocardiography at our hospital’s Cardiology Service. Diabetic patients showed higher CIMT compared to healthy controls, with this difference being identified in the left carotid (*p* < 0.001) (Figure 3A) and right carotid (*p* = 0.003) (Figure 3C).

We observed that the poorly controlled diabetic population had significantly higher left CIMT than the well-controlled diabetic group (*p* = 0.024) (Figure 3B). On the other hand, right CIMT proved to be significantly higher in the poorly controlled diabetic group than in the control group (*p* = 0.001) (Figure 3D).

### 3.5. Correlation Analysis

**Figure 4:**
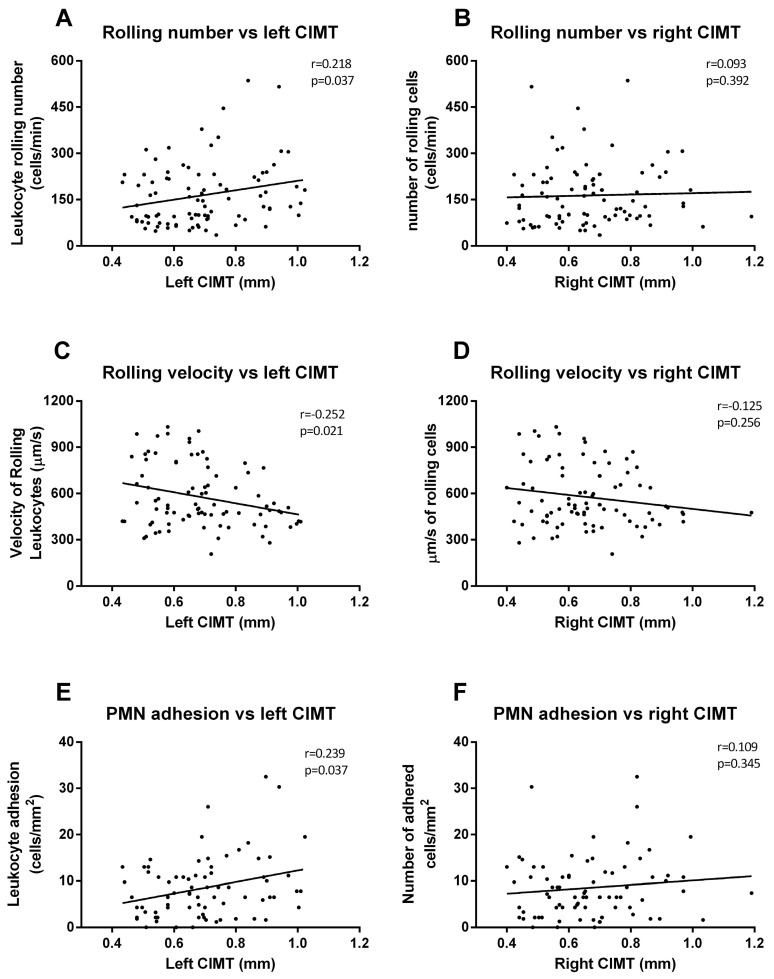
**Correlation graphs of adhesion assay vs. CIMT measures**. Graphs show correlations between number of rolling PMN and left (**A**) and right CIMT (**B**); rolling velocity and left (**C**) and right CIMT (**D**); and cell adhesion and left (**E**) and right CIMT (**F**). Spearman correlation analysis was performed.

We took all these data and performed correlations and linear regression to explore relations between all the analyzed variables. First, we analyzed the relationship between in vitro adhesion assay parameters and the left CIMT; we observed positive correlations (rolling number Figure 4A, *p* = 0.037, r = 0.218; rolling velocity Figure 4C, *p* = 0.021, r = 0.252; adhesion Figure 4E, *p* = 0.037, r = 0.239) among the left carotid measures but not among those of the right (Figure 4B,D,F).

**Figure 5:**
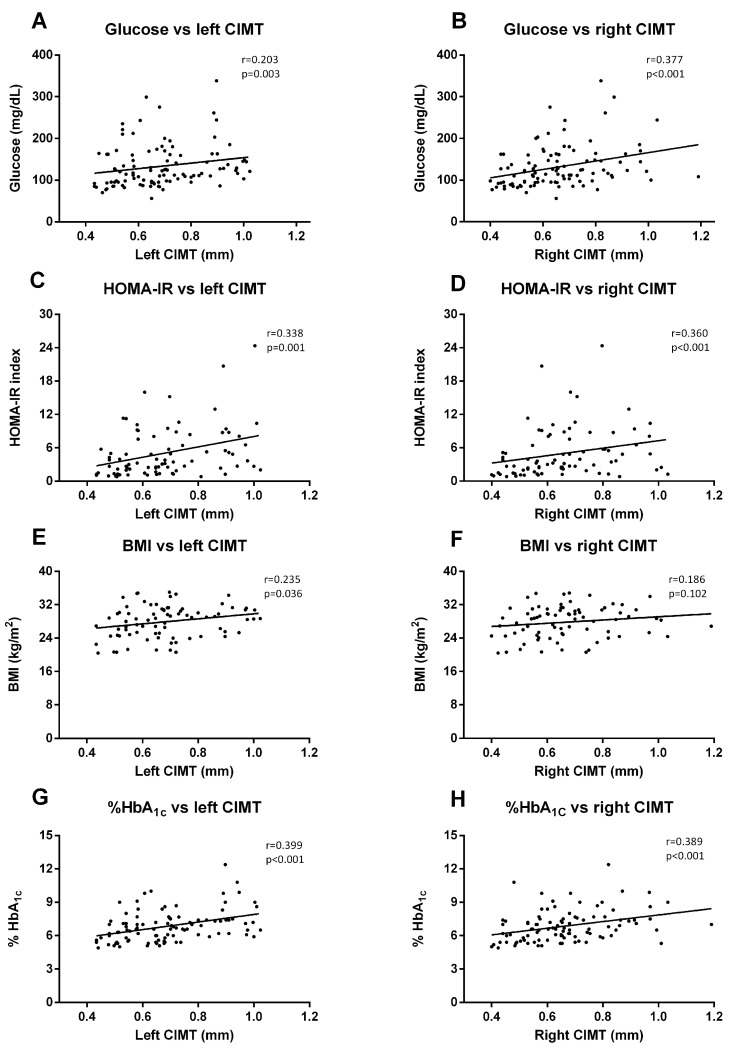
**Correlation graphs of biochemical and anthropometrical parameters vs. CIMT measures.** Graphs show correlation between glucose levels and left (**A**) and right CIMT (**B**); HOMA index and left (**C**) and right CIMT (**D**); BMI and left (**E**) and right CIMT (**F**); and HbA_1c_ and left (**G**) and right (**H**) CIMT. Spearman correlation analysis was performed.

Regarding biochemical parameters, we saw that left CIMT measures correlated significantly with glucose (*p* = 0.003, r = 0.203) (Figure 5A), HOMA-IR (*p* < 0.001, r = 0.338) (Figure 5C), BMI (*p* = 0.036, r = 0.235) (Figure 5E), and HbA1c (*p* < 0.001, r = 0.399) (Figure 5G). These correlations were similar for the right CIMT, except for BMI correlation, which was not significant (Figure 5B (Glucose), *p* < 0.001, r = 0.377; Figure 5D (HOMA-IR), *p* < 0.001, r = 0.360; Figure 5F (HbA1c), *p* < 0.001, r = 0.389).

**Figure 6:**
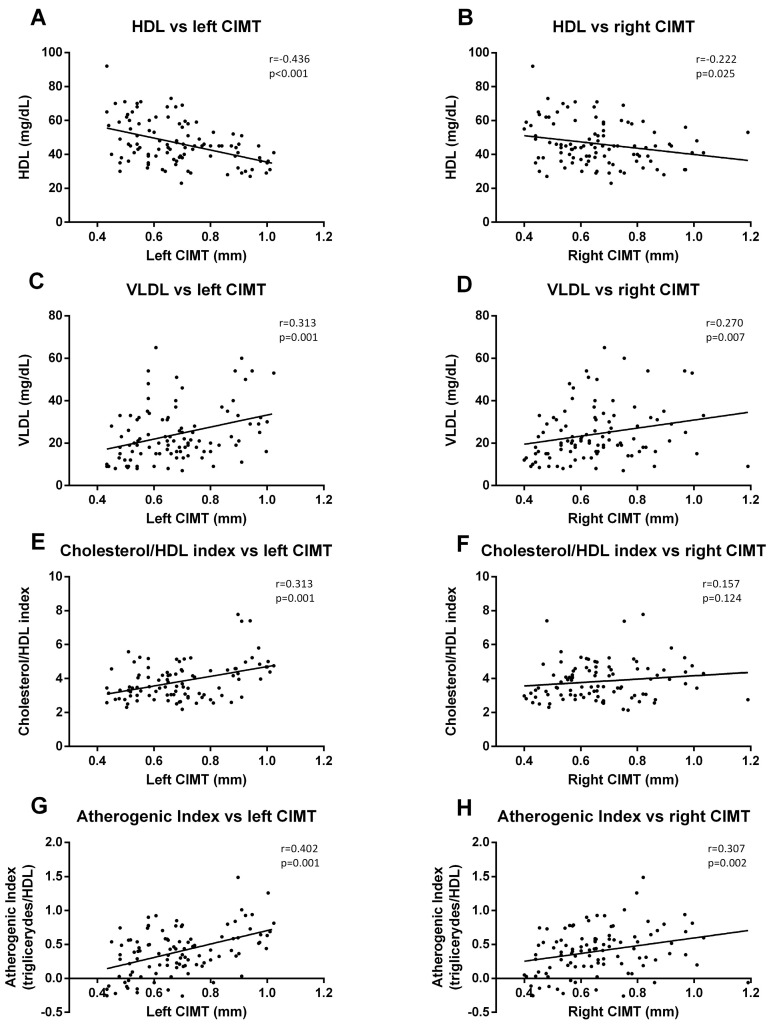
**Correlation graphs of lipid metabolism parameters vs. CIMT measurements**. Graphs show a correlation of HDL-c with left (**A**) and right CIMT (**B**); of VLDL index with left (**C**) and right CIMT (**D**); of cholesterol/HDL-c index with left (**E**) and right CIMT (**F**); and of atherogenic index (AIP) with left (**G**) and right (**H**) CIMT. A Spearman correlation analysis was performed. r coefficient and statistical significance, if any existed, are shown in the graph.

When we analyzed the correlation with lipid parameters, we observed that left CIMT was significantly correlated with HDL-c (*p* < 0.001, r = −0.436) (Figure 6A), VLDL (*p* = 0.001. r = 0.313) (Figure 6C), cholesterol/HDL index (*p* = 0.001, r = 0.313) (Figure 6E), and AIP (*p* = 0.001, r = 0.402) (Figure 6G). The data for right CIMT revealed similar correlations (Figure 6B (HDL-c), *p* = 0.025, r = −0.222; Figure 6D (VLDL), *p* = 0.007, r = 0.270; Figure 6H (AIP), *p* = 0.002, r = 0.307), with the exception of cholesterol/HDL index correlation.

## 4. Discussion

Cardiovascular complications are a principal concern during diabetes management. The present study gives relevance to the relationship between CIMT, HbA_1c_, and different hallmarks of T2D (inflammation, ROS production, and leukocyte–endothelium interactions). We have evaluated the involvement of glycemic control in endocrine and anthropometric parameters, inflammatory markers (TNF-α, IL-6, and mtROS production), adhesion molecules (ICAM-1, VCAM-1, and P-selectin), leukocyte–endothelium interactions (rolling, rolling velocity, and adhesion) and CIMT in T2D. In addition, we have analyzed their interrelationship by performing correlation studies. T2D patients, and especially those with poor glycemic control (HbA_1c_ > 6.5%), expressed an increase in inflammatory markers, mtROS production, adhesion molecules, leukocyte–endothelium interactions, and CIMT.

Regarding inflammatory intermediates, our study shows a slight but significant increase in TNF-α and IL-6 production in T2D patients; it is possible that the difference is not bigger because of the hypolipemiant treatments received by most of the T2D patients. These proinflammatory cytokines are involved in the development of inflammation in T2D. Enhanced levels of TNF-α from leukocytes after activation by ROS-induced oxidative stress are thought to impair glucose uptake and inhibit insulin signaling [70,71]. Furthermore, IL-6 is thought to play an important role in atherosclerosis in T2D [56]. We show an increase in mtROS production in leukocytes from T2D patients that was more pronounced in subjects with HbA_1c_ > 6.5%, suggesting that leukocyte mitochondrial function can be altered during chronic hyperglycemia [70,72,73,74]. Other studies in the field have suggested that good glycemic control reduces ROS production [57,75,76]. These results are in accordance with those of other studies that have demonstrated high mtROS production in T2D related to the development of silent myocardial ischemia [72]. In this sense, it is important to underline that leukocytes are especially linked to ROS generation and cells that are highly sensitive to the oxidative damage mediated by ROS [77,78].

Different pathophysiological processes, including hypertension and atherosclerosis, are characterized by leukocyte recruitment to the arterial wall. In the present study, we have used an in vitro model in which human leukocytes flow over a monolayer of human endothelial cells with a shear stress similar to that observed in vivo [72]. This mimics the process that precedes inflammation in vivo (rolling and adhesion), and which is critical to homeostasis and vascular cell integrity. Our experimental system has been widely applied to visualize and analyze the multistep recruitment of leukocytes in these diseases, and allows the mechanisms of action implicated in this recruitment to be assessed [79]. Regarding this idea, it has been demonstrated that an inflammatory background favors the increase of leukocyte–endothelium interaction and promotes the early development of atherosclerotic events [80,81]. In the current study, we have observed that T2D enhanced rolling flux and PMN adhesion and reduced the rolling velocity of PMN. These effects were more evident in the group with HbA_1c_ > 6.5%. Furthermore, several studies have demonstrated the importance of leukocytes in the atherosclerotic scenario [82,83,84]. In accordance with these results, an increase in leukocyte–endothelium interactions has been related to oxidative stress in a human model of insulin resistance [85]. In addition, Petterson et al. demonstrated that there is increased recruitment but impaired function of leukocytes during inflammation in mouse models of T1D and T2D [86].

Endothelial and immune cell activation can be evaluated by measuring the soluble adhesion molecules VCAM-1, ICAM-1, and P-selectin. In this sense, it has been described that adhesion molecules are enhanced in patients with T2D [87]. In the present study, we show an increase in adhesion molecules, ICAM-1 and VCAM-1, in T2D that was most pronounced in the case of VCAM-1 in the HbA_1c_ > 6.5% group. These results are compatible with a rise in the number of leukocyte–endothelium interactions, and it has been demonstrated that hyperglycemia in both normal subjects and T2D patients can induce vasoconstriction, adhesion molecules, and inflammation [88,89]. Importantly, there was a slight but significant increase of T2D adhesion molecule levels with respect to the control group, a difference that may have been reduced by the hypolipemiant treatment.

The measurement of CIMT is useful for monitoring the early stages of atherosclerosis [61,90], and CIMT enhancement has been described in T2D [91]. In the present study, we have observed an increase in left and right CIMT, especially in the former case. Furthermore, the increment was more evident in the HbA1c > 6.5% group, suggesting that glycemic control is crucial for leukocyte–endothelium interactions and, therefore, for CIMT. The relevance of these changes in the left CIMT remain to be clarified, though different studies have suggested variations between left and right carotids; for example, Lorentz M. W. et al. revealed that left carotid plaques were vulnerable, whereas right carotid ones were calcified and stable [65]. Luo X et al. studied the factors associated with left and right CIMT and found that changes in biochemical parameters were associated with left carotid measures, while hemodynamic parameters were more related to right carotid measures. [92]. The main consequences of CIMT thickening are cerebrovascular events such as stroke, and left carotid stroke is more frequent because of a higher probability of thickening of the left carotid arterial wall [93,94]. The above mentioned authors highlighted that the location of the left carotid renders it more susceptible to hemodynamic stress, thus increasing the probability of arterial wall thickening and rupture. Selwaness M. et al. support this hypothesis; they found that while bilateral plaques were more frequent, 67% of cases of unilateral plaque occurred in the left carotid. This left plaque presented more intraplaque hemorrhage and more fibrous tissue and was thicker than the left, all of which explain why the left plaque is more vulnerable and prone to stroke. In the same study, right CIMT was found to be more calcified than the left, which would make it more resistant to shear stress [95].

In the present study, we have observed positive correlations between in vitro adhesion assay parameters and left CIMT, but not right CIMT. These results confirm the relevance of the enhancement of leukocyte–endothelium interactions in CIMT, especially on the left side. In terms of biochemical parameters, left CIMT measures correlated significantly with glucose, HOMA-IR, BMI, and HbA_1c_. These correlations were maintained in the right CIMT, except for BMI (which was not significant). In line with these results, a systematic review by Einarson et al. found that individuals with impaired glucose tolerance had slightly (though significantly) higher CIMT values than individuals with normal glucose tolerance [96]. This data, together with leukocyte–endothelium interactions, suggest that poor glycemic control leaves T2D diabetic patients more prone to developing early or subclinical atherosclerotic events due to the rise in the number of leukocytes infiltrating the intima–media layer.

Finally, we analyzed correlations between CIMT and lipid parameters, and observed that the left CIMT was significantly correlated with VLDL, cholesterol/HDL index, and AIP. All these correlations were maintained when we analyzed the right CIMT data, with the exception of the cholesterol/HDL index correlation. In this sense, [97] demonstrated that lipid parameters, including total cholesterol (TC), triglycerides (TG), LDL, and VLDL, were significantly higher in diabetic stroke patients and positively correlated with the risk of stroke. CIMT was significantly higher in diabetic stroke patients, and correlations of lipid parameters (TC, TG, and VLDL) with CIMT in said patients were significantly and positively correlated, while lipid parameters (TC, TG, HDL, and LDL) were negatively correlated in nondiabetic ischemic stroke patients. Although lipidic parameters were differently affected by glycemic control, it is clear that these parameters increase the risk of developing later cardiovascular complications by increasing CIMT. We did not find any significant correlation with mitochondrial ROS production, adhesion molecules, or cytokine concentrations, though there was a tendency toward a slight correlation.

This study is observational, and so it would be interesting to perform a longitudinal intervention study in which we assess the evolution of CIMT in patients with poor glycemic control that achieve a good glycemic control. Defining the reason why left and right carotids behave and are affected differently is still unclear, and further research focusing on this issue would be useful. Moreover, we have correlated many T2D markers with one indicator of cardiovascular risk; future studies could attempt to find a correlation with other cardiac and endothelium function markers to reinforce our findings.

## 5. Conclusions

The current study provides evidence of proinflammatory markers, mtROS production, leukocyte–endothelium interactions, adhesion molecules, and CIMT in T2D. Some of these alterations were more pronounced in patients with HbA1c > 6.5, suggesting that glycemic control is a useful tool for preventing or delaying the onset of subclinical atherosclerotic process. Future research into these aspects will help to clarify the molecular mechanisms involved in glycemic control in T2D, and to modulate and control the atherosclerotic process in T2D.

## Figures and Tables

**Table 1:** Anthropometrical and biochemical parameters.

	Control	T2D	
		HbA_1c_ ≤ 6.5%	HbA_1c_ > 6.5%
**N**	108	57	104
**Age (Years)**	57 ± 11	58 ± 8	60 ± 9
**%Women**	62.2%	43.93%	56.11%
**Weight (kg)**	68.51 ± 15.18	85.02 ± 16,07 *	83.59 ± 15.84 *
**BMI (kg/cm^2^)**	24.18 ± 4.11	31.18 ± 4.23 *	30.43 ± 5.13 *
**SBP (mmHg)**	119.43 ± 18.18	139.82 ± 14.23 *	138.38 ± 17.05 *
**DBP (mmHg)**	72.35 ± 10.94	82.03 ± 10.97 *	78.25 ± 9.35 *
**Waist (cm)**	79.81 ± 12.62	106.39 ± 11.46 *	103.99 ± 13.35 *
**Hip (cm)**	99.09 ± 7.21	108.71 ± 9.53 *	108.48 ± 12.62 *
**Waist–Hip ratio**	0.80 ± 0.09	0.97 ± 0.08 *	0.95 ± 0.08 *
**Glucose (mg/dL)**	88.08 ± 10.75	112.37 ± 22.59 *	160.51 ± 55.06 *^‡^
**HOMA**	1.69 ± 1.19	3.72 ± 2.00 *	6.57 ± 4.45 *^‡^
**HbA_1c_ (%)**	5.18 ± 0,26	5.94 ± 0.30 *	7.85 ± 1.30 *
**Total cholesterol (mg/dL)**	185.43 ± 35.32	173.67 ± 34.31	167.42 ± 37.67 *
**HDL-c (mg/dL)**	56.04 ± 13.61	45.10 ± 11.83 *	42.94 ± 10.46 *
**LDL-c (mg/dL)**	111.38 ± 28.72	102.62 ± 31.33	95.17 ± 31.09 *
**VLDL (mg/dL)**	26.01 ± 10.81	28.63 ± 19.54 *	28.91 ± 22.04 *
**Cholesterol/HDL**	3.46 ± 0.94	4.07 ± 1.18 *	4.06 ± 1.14 *
**Triglycerides (mg/dL)**	87.62 (55.50;103.00)	130.29 (90.5; 169.00) *	150.75 (92.00; 162.63) *
**Non-HDL Cholesterol (mg/dL)**	129.39 ± 33.28	129.37 ± 32.51	124.48 ± 36.57
**AIP (TG/HDL-c)**	0.10 (−0.06; 0.33)	0.47 (0.23; 0.63) *	0.47 (0.29; 0.68) *
**APO A1 (mg/dL)**	164.02 ± 32.28	151.45 ± 27.21 *	142.72 ± 22.87 *^†^
**APO B (mg/dL)**	90.78 ± 26.60	90.33 ± 25.82	94.18 ± 25.27
**APOB/APOA1**	0.57 ± 0.20	0.64 ± 0.24	0.67 ± 0.19 *
**hsCRP (mg/L)**	0.75 (0.36; 1.83)	2.64 (1.61; 7.07) †	2.87 (1.31; 6.59) †

Anthropometrical and biochemical parameters obtained from whole peripheral blood from healthy subjects, HbA_1c_ ≤ 6.5% T2D patients and HbA_1c_ > 6.5% T2D patients after 12 h fasting. Kolmogorov–Smirnov normality test was performed in all data sets. Data are shown as mean ± SD for data with normal distribution, and median and 25th; 75th percentile for non-normal data. The differences were analyzed by a t-test in the case of normal data and a Mann–Whitney test in that of non-normal data. *, *p* < 0.01 vs. control; †, *p* < 0.001 vs. control; ‡, *p* < 0.001 vs. T2D HbA_1c_ ≤ 6.5%.

## Supplementary Materials

The following are available online at https://www.mdpi.com/2077-0383/9/8/2522/s1, Table S1: Time of T2D evolution on the recruited patients, Table S2: Incidence of most common comorbidities in the recruited T2D patients and in the control population, Table S3: Prescription of the different treatments associated with T2D in all the study subjects, Table S4: Leukocyte composition in healthy subjects and diabetic patients., Video S1: Control, Video S2: HbA1c > 6.5, Video S3: HbA1c ≤ 6.5.

## Abbreviations

ADA	American Diabetes Association
AGE	Advanced glycation end products
AIP	Atherogenic index of plasma
CIMT	Carotid intima–media thickness
CT	Cholesterol
DAPI	4′6′Diamidin-2-fenilindol
DBP	Diastolic blood pressure
HbA_1c_	Glycated hemoglobin
HBSS	Hanks’ Balanced Salt Solution
HDLc	High-density lipoprotein cholesterol
HOMA-IR	Homeostatic model assessment–insulin resistance
HUVEC	Human umbilical cord vein endothelial cell
ICAM-1	Intercellular adhesion molecule 1
IL-6	Interleukin 6
IR	Insulin resistance
LDLc	Low-density lipoprotein cholesterol
PMN	Polymorphonuclear cells
ROS	Radical oxygen species
RPMI	Roswell Park Memorial Institute culture medium
SBP	Systolic blood pressure
TG	Triglycerides
TNFα	Tumor necrosis factor alpha
VCAM-1	Vascular cell adhesion molecule 1
VLDLc	Very low-density lipoprotein cholesterol
